# I’m Walking into Spiderwebs: Making Sense of Protein–Protein Interaction Data

**DOI:** 10.1021/acs.jproteome.3c00892

**Published:** 2024-04-01

**Authors:** Chase L. S. Skawinski, Priya S. Shah

**Affiliations:** 1 Department of Chemical Engineering, University of California, Davis 95616, California, United States; 2 Department of Microbiology and Molecular Genetics, University of California, Davis 95616, California, United States

**Keywords:** protein−protein interactions, mass spectrometry, proteomic scoring

## Abstract

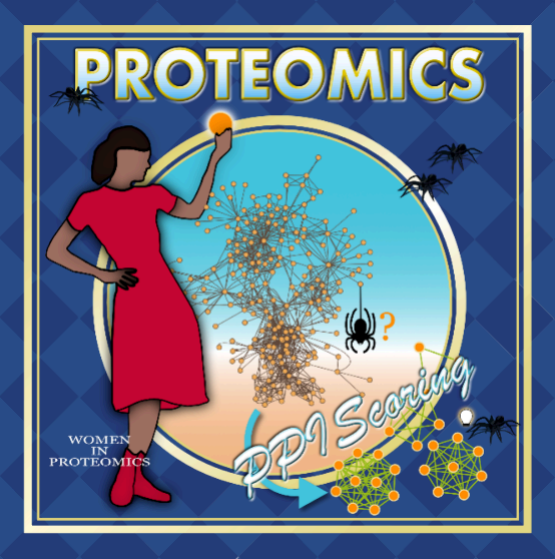

Protein–protein interactions (PPIs) are at the heart of the molecular landscape permeating life. Proteomics studies can explore this protein interaction landscape using mass spectrometry (MS). Thanks to their high sensitivity, mass spectrometers can easily identify thousands of proteins within a single sample, but that same sensitivity generates tangled spiderwebs of data that hide biologically relevant findings. So, what does a researcher do when she finds herself walking into spiderwebs? In a field focused on discovery, MS data require rigor in their analysis, experimental validation, or a combination of both. In this Review, we provide a brief primer on MS-based experimental methods to identify PPIs. We discuss approaches to analyze the resulting data and remove the proteomic background. We consider the advantages between comprehensive and targeted studies. We also discuss how scoring might be improved through AI-based protein structure information. Women have been essential to the development of proteomics, so we will specifically highlight work by women that has made this field thrive in recent years.

## Introduction

Coordinated interactions between proteins mediate a myriad of cellular functions throughout life. These protein–protein interactions (PPIs) can occur directly between two proteins, but many comprise multiple proteins that work together as a complex to execute specific functions. Protein complexes form as quaternary structures from the non-covalent interactions of multiple proteins via hydrogen bonds and van der Waals forces. Studying PPIs can reveal mechanisms of cellular homeostasis,^1,2^ dynamic cellular processes (e.g., cell signaling, division, or differentiation),^3^ and disease.^4,5^ Here, we review how PPIs can be identified using mass spectrometry (MS)-based approaches, the caveats associated with analyzing such data rigorously, and the opportunities for future innovation in the field. Instead of providing a comprehensive review of such a broad field for this special issue focused on women in proteomics, we highlight work by women when possible (see notes).

## MS-Based Approaches in PPI Identification

MS identifies proteins using unique mass spectra, or mass-to-charge ratio fingerprints. This can be done using a “bottom-up” or “top-down” method. Bottom-up proteomics requires enzymatic digestion of a protein sample into peptide fragments, while top-down proteomics will use whole proteins or large fragments. In both cases, peptides (for bottom-up) or intact proteins (for top-down) are separated by liquid chromatography (LC) prior to ionization and fragmentation for MS analysis. For top-down proteomics, intact proteins can undergo size separation prior to LC-MS, which helps the mass spectrometer to identify proteins or proteoforms in each sample. Many groups have recently published reviews on these topics, including a bottom-up review by Plubell et al.,^6^ a top-down review by Po and Eyers,^7^ and a general mass spectrometry review by Shuken.^8^ Data-dependent acquisition (DDA) is often the focus of reviews because of its simplicity and wide use as an MS data acquisition technique. However, data-independent acquisition (DIA) has gained popularity due to its increased sensitivity in detecting lower-abundance peptides. This is possible because of computational advancements that identify peptides from complex mixtures containing multiple precursor ions. DIA MS was recently reviewed by Ludwig et al.^9^ and Zhang et al.^10^

Regardless of the method selected, once mass spectra are acquired, they are mapped back to proteins based on a peptide search against the organism of study. To perform the mapping process, computational analysis software, such as DIA-NN,^11^ MSFragger,^12^ Skyline,^13^ MaxQuant,^14^ Protein Prospector,^15^ Spectronaut,^16^ and many more, will map the spectra to peptides that theoretically match based on known amino acid sequences. The software then maps the peptides to proteins. Since spectra mapping to peptides can give false positives, decoy peptides, created through a variety of computational techniques, can be used to estimate the false discovery rate (FDR) and ensure it is acceptably low.

Variations on LC-MS provide varying degrees of resolution in PPI identification, depending on the study’s goals. Methods available include classic affinity purification–mass spectrometry (AP-MS), cofractionation mass spectrometry (CF-MS), thermal proximity coaggregation (TPCA), and cross-linking mass spectrometry (XL-MS). Here, we provide brief explanations of these four approaches while discussing the advantages and caveats of each (Table 1).

**Table 1:** Summary of Mass Spectrometry Approaches With Associated Benefits and Limitations of Each Approach

approach	benefits	limitations
AP-MS	modular and scalable for use with multiple baits, can be used with proximity labeling for spatially and temporally resolved PPIs	protein interactions that are not biologically relevant may occur after cell lysis during standard AP-MS, unstable interactions may not be detected, direct vs indirect interactions and specific protein complexes cannot be distinguished
CF-MS	can identify proteins participating in multiple complexes, can be combined with AP approaches to increase resolution	protein interactions that are not biologically relevant may occur after cell lysis, protein complexes can be obscured by the co-fractionation of different complexes at the same time
TPCA	directly identifies protein complex members, compatible with multiplexed labeling to increase throughput	experimental conditions may alter melt temperatures, extensive pre-work may be required to generate melt curves
XL-MS	proteins and interactions are fixed *in situ* prior to lysis, improved protein complex identification over traditional AP-MS	crosslinked peptides require specialized analysis and FDR management

As the name implies, AP-MS experiments rely on affinity purification of a protein of interest or “bait”. Purification can involve immunoprecipitation using antibodies against the native protein or expressing bait fusions with an affinity tag (e.g., FLAG, HA, His, Strep, etc.). While purifying native proteins using specific antibodies preserves relevant biology (e.g., expression patterns), affinity tagging provides an attractive level of modularity for large-scale studies. Regardless of approach, the bait protein is purified from cell lysates using antibody-binding or affinity-tag-binding beads. LC-MS then identifies the interacting proteins, or “prey”. The flexibility and modularity of AP-MS make it a valuable approach, and many groups have applied AP-MS to make major PPI discoveries across a broad range of biology, including ion channel function in the mammalian brain,^17^ chromatin remodeling in plants,^18^ and influenza A virus replication.^19^ When conducted on a large scale, they can be used to map PPIs across an entire proteome^20^ and infer stoichiometries of protein complexes.^21^ Despite the advantages of AP-MS, there are several caveats to the approach. The sample lysis and purification can introduce background protein binding through the mixing of cellular compartments that are usually isolated in space and/or time. Detergents in lysis buffers can also disrupt transient and/or weakly binding proteins and protein complexes and may bias the data to PPIs with higher binding affinities.

One extension of the AP-MS approach that deals with many caveats of classic AP-MS is proximity labeling. This approach takes advantage of bait fusion with an enzyme that can label neighboring proteins with biotin moieties *in situ*. Subsequent AP-MS of the biotinylated proteins can define protein neighborhoods or complexes. Proximity labeling can help maintain low-affinity or transient interactions that would be lost by AP-MS alone. By purification of proteins labeled *in situ* instead of from lysates, proximity labeling avoids mixing proteins from different compartments or cell states that could give rise to spurious interactions. However, proximity labeling is also subject to off-target background labeling, and careful controls must be included to account for this.^22,23^

While many proximity labeling tools have been developed, we highlight technologies developed by Alice Ting’s group and applied to identify PPIs and their dynamics.^23−27^ APEX^23^ (ascorbic acid peroxidase) and the related APEX2^25^ catalyze the formation of biotin phenoxyl-radicals that spontaneously react with (primarily) tyrosine residues on timescales that limit diffusion to less than 20 nm. TurboID catalyzes direct biotinylation of target proteins through its biotin ligase activity.^24^ Compared to its BioID precursor,^28^ the engineered TurboID reaction kinetics are improved by orders of magnitude and are much closer to APEX2 labeling kinetics. Ting and collaborators have also created variations on these tools, including split versions to directly assay PPI-dependent labeling,^26^ and smaller enzymes that may be better tolerated as fusions.^24^ Though not as mature as traditional AP-MS, proximity labeling combined with AP-MS has already been used for large-scale PPI mapping efforts.^29^ Taken together, AP-MS is a powerful MS-based technique to identify PPIs because it is straightforward, accessible, and modular, although technological innovations continue.

In CF-MS, samples undergo separation using size exclusion chromatography or other techniques.^30−32^ CF-MS identifies proteins in each discrete fraction and links them together as members of a larger complex. Proteins can often take part in multiple complexes, or a complex can have different modifying members to give it unique functions. CF-MS enables efficient identification of these ensembles compared to AP-MS. However, samples are still lysed in solution prior to fractionation, which can bias detection toward complexes stable under specific lysis conditions. Multiple complexes may also cofractionate together, making data validation important. AP can also be added as an intermediate step to further reduce complexity or identify complexes with specific members.^31^ Andrea Fossati and colleagues recently applied CF-MS to identify jumbophage-bacteria PPIs.^33^ Jumbophages encode more than 300 proteins, and identifying PPIs using AP-MS is not necessarily practical. By using CF-MS, the authors were able to compare jumbophage-bacteria PPIs across two jumbophages and found evidence of shared phage predation mechanisms between the two viruses. While CF-MS is still relatively new, it holds promise for many systems.

TPCA was developed by Chris Tan and colleagues as a high-throughput method to separate interacting proteins based on denaturation temperature.^34^ This technique is a unique application stemming from the cellular thermal shift assay (CETSA) developed in 2013 by Martinez Molina et al.^35^ and thermal proteome profiling (TPP) developed in 2014 by Savitski et al.,^36^ originally used to characterize melting point shifts in proteins and protein complexes during ligand or drug binding.^37^ TPCA uses MS and multiplexed quantification of the CETSA to generate melt curves for protein complexes. Since protein complexes will denature at a similar temperature and coaggregate, TPCA can identify proteins with similar melt curves and solubility behaviors to assign them to protein complexes. In an elegant application of TPCA, Joshua Justice and colleagues identify global changes in PPIs caused by herpesvirus infection.^38^ The authors show that DNA sensor IFI16 (interferon gamma inducible protein 16) recruits DNA-PK (DNA-dependent protein kinase) early during infection. DNA-PK phosphorylates IFI16 on tyrosine 149 to initiate a cytokine response. Although TPCA can be a powerful tool, cell lysis and other manipulations of the sample can change the proteins’ environments and alter their thermal stabilities. Each system of interest may require new calibrated melt curves based on the experimental conditions. The computational analysis is also not trivial. Consequently, this method has not been applied as broadly as the others. Nonetheless, TPCA is far more scalable in identifying PPIs across the entire proteome and for many different conditions.^39^

XL-MS^40^ uses a crosslinking reagent to create a covalent bond between adjacent peptides and can be combined with AP-MS (XL-AP-MS) to identify PPIs. When crosslinking occurs between neighboring proteins within a complex, this can stabilize protein complexes and even provide structural information about points of contact between the proteins. Reversible crosslinking mainly serves to stabilize transient interactions. Irreversible crosslinking will alter the spectra produced since the peptides remain covalently bonded for LC-MS analysis and can be used to identify which peptides are in contact. However, spectra derived from samples without reverse crosslinking must be mapped using specialized databases and algorithms, and the FDR calculation is not as straightforward. A recent large-scale study by Swantje Lenz and colleagues applied XL-MS to *Escherichia coli* lysates to recover 590 PPIs. The authors also demonstrated the power for structural discovery by successfully mapping the interaction domain between an uncharacterized protein YacL and RNA polymerase using the crosslinking data.^41^ A more focused study by Sara Ayala Mariscal and colleagues used XL-MS to study the huntingtin protein (HTT), whose expanded polyglutamine (polyQ) tracts cause the neurodegenerative Huntington’s Disease through HTT aggregation. The authors mapped a specific binding motif between HTT and chaperone DNAJB1 (DnaJ heat shock protein family member B1) using XL-MS. These results were used to target mutations to this binding motif and disrupt the interaction.^42^ Given the advances in protein structure prediction and cryo-electron microscopy, XL-MS is an incredibly promising method to rapidly go from large-scale datasets down to amino acid resolution of PPIs.^43^

## You’ve Got Me for Your Prey: PPI Data Curation and Scoring Tools

**Figure 1:**
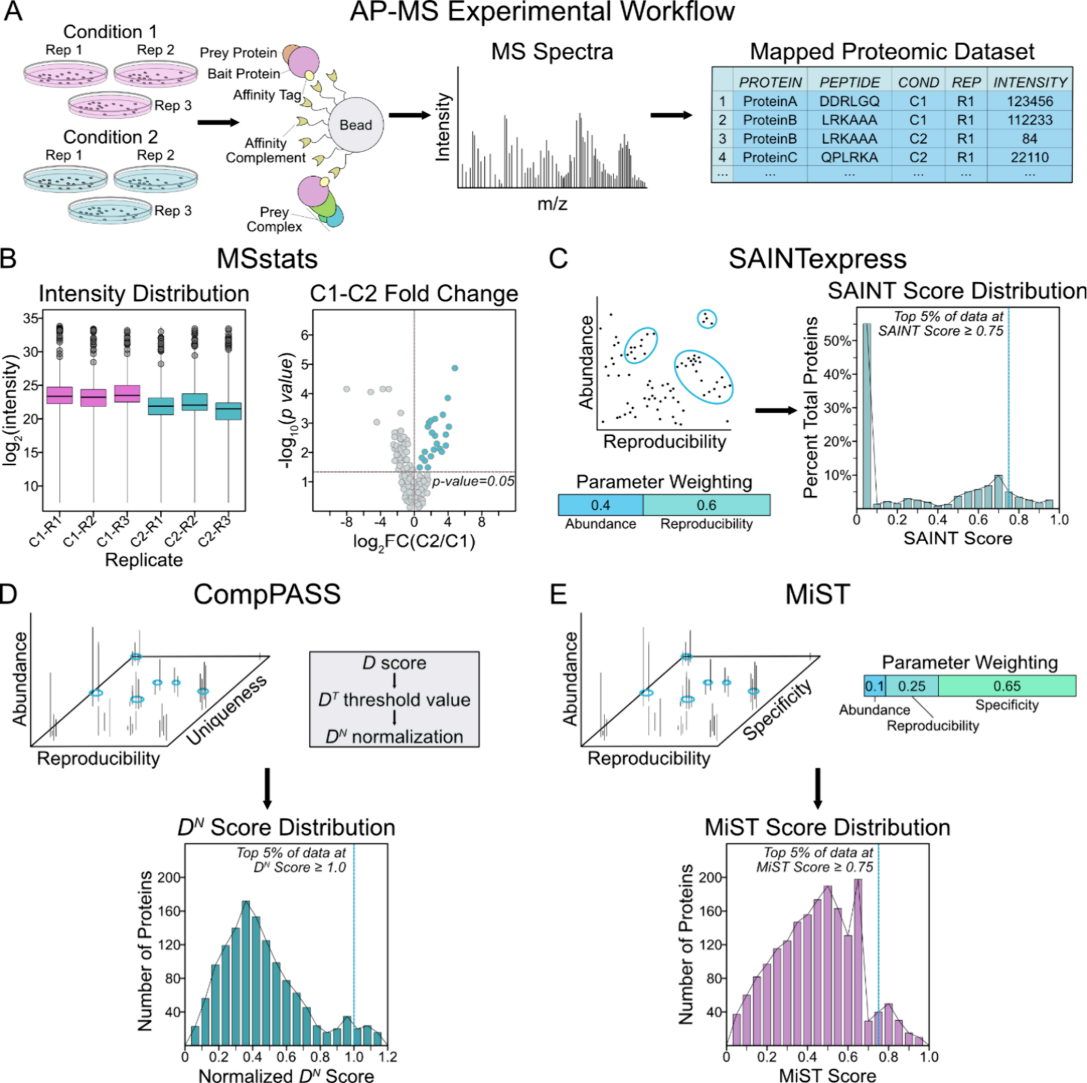
Brief explanations of four methods used when evaluating affinity purification–mass spectrometry (AP-MS) datasets. (A) A general experimental AP-MS workflow. Two conditions (C1 and C2) each with three replicates (R1, R2, and R3) are depicted. Samples undergo purification and mass spectrometry, where the peptide data are recorded and mapped to known proteins. (B) An example of MSstats outputs for an AP-MS dataset. MSstats can provide relative statistical quantitation for data in the form of a box plot showing data distribution across all conditions and replicates and a volcano plot showing fold change for proteins among the two conditions. Blue dots indicate proteins with a positive log-2 fold change and a *p*-value below 0.05 as an example of a high-confidence PPI. (C) Example of the SAINT and SAINTexpress output. Parameters for abundance and reproducibility are weighed against the dataset to provide a score associated with the presence of prey proteins in each replicate. (D) Example of the CompPASS output. The algorithm evaluates prey abundance, reproducibility, and uniqueness through all replicates and calculates a *D* score for each prey based on these criteria. A normalized *D* score plot is shown. (E) Example of MiST output for virus–host PPI data. MiST generates scores using the same three criteria as CompPASS, but the weighting for each criterion is set by the user.

After completion of the peptide mapping process (Figure 1A), the output dataset may contain tens or thousands of prey, not all of which indicate real or biologically relevant interactions with the bait(s). Combine this with the background contaminants that find their way into samples (e.g., hair keratins and bacterial proteins), and it becomes obvious that researchers could easily be stuck in a PPI web that mass spectrometry is spinning. Consequently, they need an unbiased method to remove these extraneous or irrelevant prey from their datasets. Some groups keep personal databases of known background and contaminant proteins while others use freely available tools such as CRAPome (Contaminant Repository for Affinity Purification).^44^ Either way, elimination of extraneous proteins can reduce a dataset’s size and provide a starting point for confident PPI identification. This helps to make both data analysis and complex inferences more tractable.

High-confidence interactions do not stem from a single datapoint, but instead arise from holistic analysis of the entire dataset using an unbiased and systematic scoring method. These scoring methods can be used to derive a threshold or minimum performance value that a protein must reach to be considered an interactor for a certain bait. Enrichment of specific proteins under multiple conditions can be established with statistical quantification tools such as MSstats,^45−47^ a package created by Meena Choi and Olga Vitek (Figure 1B). MSstats normalizes data across runs to account for differences in total protein content and then performs direct comparisons between each condition and replicate to calculate enrichment (fold changes) and significance (*p*-values) for each protein. Statistical tools such as MSstats can perform direct comparisons and impute protein intensity or spectral counts for missing values. The user must define cutoffs for enrichment and significance to threshold their data.

Since AP-MS is still the most broadly used approach to identify PPIs, many data analysis tools have been developed to handle these datasets specifically. Algorithms and tools such as SAINT (Significance Analysis of INTeractome)^48^ or SAINTexpress,^49^ CompPASS (Comparative Proteomic Analysis Software Suite),^50^ and MiST (Mass Spectrometry Interaction STatistics)^51^ can take an AP-MS dataset and provide a numerical score for each PPI. These tools take a given dataset and review it for two or three criteria: the abundance of the protein, the reproducibility or presence of a PPI across multiple replicates, and the specificity or uniqueness of an interaction (for MiST and CompPASS only).

The SAINT/SAINTexpress^48,49^ algorithms perform quantitative data analysis and probabilistic scoring of a proteomic dataset as a means of evaluating the likelihood of a bait–prey pair interaction (Figure 1C). In its original form, SAINT used only the quantitative data associated with each prey’s total spectral counts recorded in a bait condition and compared them against all baits and their replicates. Negative controls were not required if sufficient independent bait conditions were profiled together, permitting analysis of both small and large datasets. Using statistical modeling, a prey’s counts in a single bait are modeled from a Poisson distribution representing either a true interaction or a false interaction. Distributions are calculated for each bait–prey pair throughout the dataset in a matrix, resulting in a probability associated with each PPI. The probabilities help to determine a Bayesian FDR and a threshold probability needed to achieve it. SAINTexpress expanded upon SAINT with the ability to perform calculations using protein intensity values in addition to spectral counts. It also contains information on existing PPI data, allowing it to supplement the main score with a separate topology-based score, the topology-aware average probability score (TopoAvgP), which can improve the identification of copurifying prey–prey protein complexes.

CompPASS^50^ operates similarly to SAINT and SAINTexpress in that both will automatically output PPI probability values for each bait–prey pair in a dataset (Figure 1D). The CompPASS algorithm calculates two scores for the bait–prey pairs: a *Z* score to normalize and center the data and a *D* score based on the adjustment of total spectral counts (TSCs) across all bait conditions and replicates. The *D* score incorporates prey uniqueness under bait conditions, protein abundance (as TSC), and reproducibility across replicates to create a representative score for the three attributes. CompPASS then calculates a threshold *D* score (*D*^T^) to ensure 95% (or other configurable amount) of the data falls below this threshold. Raw *D* (*D*^R^) scores are normalized against *D*^T^, producing normalized *D* scores (*D*^N^) that can be plotted. Preys with a *D*^N^ ≥ 1.0 are considered high-confidence PPIs. If preservation of a low-scoring PPI is desired, the *Z* score can provide evidence for further validation.

MiST^51^ uses the mapped proteomic data to generate scores for specificity, reproducibility, and abundance (Figure 1E). Each of these scores is then combined in a linear combination to produce an overall MiST score. The weighting parameters for each of the three components sum to 1.0 and are typically weighted more toward specificity and reproducibility (specificity ∼65%, reproducibility ∼25%, abundance ∼10%). The emphasis on specificity emerges from the fact that MiST was originally developed to score virus–host PPIs for RNA viruses. RNA viruses have some of the smallest genomes (typically 10 kilobases or less). Evolving redundant interactions, in which multiple viral proteins interact with the same host protein, is inefficient. Consequently, the creators of MiST theorized that specificity of protein interactions would help recover gold standard virus–host PPIs.^51^ However, scoring weights can be tailored to a user’s dataset, provided there is a mechanism to evaluate the precision and recall of the tailored weights.^51,52^

Regardless of the scoring approach used, the end result will require some decision on a threshold above which PPIs are considered “high-confidence”. This will vary depending on the dataset and scoring approach but is typically set to capture the top ∼5% of scored data or optimized based on precision and recall against a gold standard dataset. A dataset originally containing thousands of potential PPIs will retain only a few (tens to hundreds) high-confidence interactors. The reality of the experiment has finally shown itself: many PPIs identified through MS do not reflect actual biological relevance due to their promiscuity or chance contact that resulted in purification. If a PPI of particular interest is known to interact with a bait and complex member but does not make it through a strict scoring threshold, systematic approaches to rescuing low-scoring interactions have been established.^53^ It is important to recognize that these scoring algorithms, while powerful in untangling spiderwebs, can evaluate only the data provided. Ultimately, refined high-confidence PPI networks are a starting point to generate hypotheses and require further testing. Next, we highlight studies that span this spectrum of hypothesis generation and testing.

## Breadth or Depth? Advantages of Comprehensive and Focused PPI Studies

Comprehensive studies of PPIs require large investments of resources, but their size can provide advantages in internal validation. For example, a recent study by André Michaelis and colleagues provided a more complete view of the *Saccharomyces cerevisiae* protein–protein interactome.^20^ The authors performed systematic purifications of nearly 4,000 green fluorescent protein (GFP)-tagged *S. cerevisiae* proteins to identify over 31,000 PPIs. This doubled the number of proteins and tripled the number of high-confidence interactions previously identified by other large-scale efforts.^54−56^ Reverse or reciprocal purifications are often used to validate specific PPIs, as Edward Huttlin and colleagues did on a large scale for BioPlex, a similar effort to map the human protein–protein interactome.^57−59^ The near complete nature of the study in *S. cerevisiae* by Michaelis and colleagues meant that almost all of these reverse purifications were already part of the dataset and could be incorporated into the approach as one of three criteria used for scoring. While not all proteins encoded by *S. cerevisiae* could be successfully tagged with GFP for purification in this study, the authors leveraged their success with other proteins to fill in the gaps presented by biochemically challenging proteins. For example, proteins from the chaperonin-containing T-complex (CCT) cannot be tagged because the tag will interfere with complex formation and function. However, the authors were able to infer CCT interactions between the eight subunits and identify novel interactions through purification of proteins that interact with one or more subunits of CCT.

As the study by Michaelis and colleagues demonstrates, the data generated by large-scale studies often drive innovation in proteomic analysis. Many of the MS scoring algorithms discussed earlier were in fact developed to systematically deal with the large PPI datasets generated for first-of-their-kind comprehensive studies. SAINT was originally designed to score yeast kinase protein interactions^48^ and has been broadly applied to PPI studies. CompPASS was originally developed to score human PPI data for a comprehensive ubiquitin ligase interaction network^50^ and has since been applied to numerous PPI studies, including virus–host PPIs. MiST was created to score virus–host PPI data for human immunodeficiency virus 1^51^ and has been applied to many other virus–host studies since.^60−63^

Other more recent studies have developed novel comparative scoring methods to identify disease-related PPIs, since the simple overlap of networks may not be sufficient to establish the presence or absence of a PPI in a specific disease-related condition. We used holistic enrichment scoring at the pathway and complex level to identify similarities and differences across flavivirus–host PPI networks.^52^ This approach highlighted a protein interaction between viral protein NS4A and the Sec61 translocon conserved across two flaviviruses and two hosts and mediated fundamental aspects of virus replication. David Gordon and colleagues developed a differential interaction score to compare three coronavirus–host PPI networks at the protein level.^53^ This approach benefits from capturing differences that fall below a strict cutoff. Another study by Danielle Swaney and colleagues took comparative protein interaction mapping even further by identifying interactions for proteins implicated in head and neck squamous cell carcinoma.^64^ In total, the authors identified protein interactions for 31 wild type and 23 mutant proteins in two head and neck cancer cell lines and one non-tumor esophageal cell line. By including a non-cancer cell line derived from a similar tissue and applying differential interaction scoring, the authors identified interactions relevant to cancer biology. For example, an interaction between Cyclin D1 (CCND1) and members of the PI3K complex was lost in cancer cells, while an interaction between fibroblast growth factor receptor 3 (FGFR3) and Daple (CCDC88C) was gained in cancer cells and activated cell migratory proteins.

Focused studies on single proteins and their interaction networks can take advantage of more complex and elegant experimental systems and proteomic analyses. Todd Greco and colleagues performed one such study on the HTT protein.^5^ The authors expressed HTT with a normal (20) or highly expanded (140) polyQ tract in mice and affinity purified the protein from young (2 months old) and aged (10 months old) brains. In this way, the authors identified 278 HTT-interacting proteins, including some that were dependent on age or polyQ expansions. They analyzed the stability of 72% of PPIs using an isotope labeling approach in tissues and found that interactions became more stable with aging. Thus, in addition to increases or decreases of specific interactions driving disease, interaction dynamics likely contribute to the dysfunction of HTT during aging. They validated 22 of these interactions by a luciferase two-hybrid, though some may interact indirectly. Finally, an elegant functional assay in a fruit fly model of Huntington’s Disease demonstrated that many of these HTT-interacting proteins modulate HTT-induced neuronal dysfunction.

While both are impressive in their own ways, the goals of a resource creation study are fundamentally different from that of a more focused mechanistic study. The scoring and follow-up validation will also differ based on these goals. In creating a comprehensive dataset that spans an entire set of proteins from a specific biological process, disease, or proteome, authors can take advantage of this scale in their validation. As stated earlier, reciprocal purifications are often built in and can even be incorporated into novel scoring methods. Additionally, scoring cutoffs for larger studies can be optimized through precision and recall analysis of validated, gold standard interactions.^51,52,65^ This approach can better identify and eliminate the proteomic background. On the other hand, smaller studies focused on a single protein can more easily sample PPIs in biologically relevant systems. However, they will have a limited ability to identify and remove proteomic background, and validation of specific interactions becomes much more important as a result.

## You Think That We Connect: Using Protein Structure and Interaction Predictions to Score PPI Data

With the emergence of AI-based structural prediction systems like AlphaFold 2 (AlphaFold)^66^ and RoseTTAFold,^67^ we can now predict protein structures and interactions with more accuracy and speed than ever before.^68^ Another algorithm, Protein Structure Transformer (PeSTo),^69^ predicts protein binding interfaces. Requiring only the protein’s structure from either experimental or predicted sources, PeSTo has shown marked accuracy when tested on a benchmark dataset. While many groups have used AlphaFold to identify potential binding sites and drive more mechanistic studies,^20,65^ there is a potential to reverse the workflow. Instead, protein interaction and binding interface scores from tools like AlphaFold, RoseTTAFold, or PeSTo could be used as an input for AP-MS scoring. For example, structure prediction could be used to refine scoring thresholds or rescue specific interactions that fall below a high-confidence threshold.

More generally, the protein structure predictions themselves could be valuable for PPI scoring. Until recently, it was computationally limiting to quickly perform structural alignment on multiple proteins. Previous sequence and structural alignment methods such as Dali,^70^ FATCAT,^71^ and TM-align (now US-align)^72,73^ performed with reliable sensitivity and accuracy but could not scale to many comparisons, limiting their application in a larger workflow. Protein structural alignment tools now incorporate neural network models to perform searches against entire structural databases or proteomes at speeds faster than ever before. In 2023, van Kempen et al. released Foldseek, which performs structural alignment for a protein against any combination of protein structures with high accuracy.^74^ Foldseek achieves this speed and accuracy in part by converting a protein’s tertiary structure into a simplified character sequence from Foldseek’s 3D interaction (3Di) alphabet. Each pair of residues closest to one another is described by one of 20 characters in this 3Di alphabet. This simplifies the alignment from a full structural alignment while adding rigor when compared with backbone-based structural alphabets or amino acid sequence alignments. When combined with the predictive power of AlphaFold and its protein structure prediction databases, Foldseek can search a query against entire proteomes and identify possible structurally homologous proteins. This could be especially valuable for PPI studies in non-model organisms, for which databases compiling experimentally resolved structures and validated protein complexes are limited. In this case, protein structure homology could be used to efficiently identify conserved protein complexes and add confidence to PPI scores when the gold standard reference interactions are unavailable.

## Conclusions

MS-based identification of PPIs is an exciting area of discovery science, but it can often feel like walking into spiderwebs. These webs can be unraveled into tractable threads using a variety of scoring approaches. Studies focused on large interactomes with many bait proteins can leverage existing PPI databases and built-in internal validation to guide scoring and thresholding. Targeted studies can employ complex experimental systems and involve more follow-up validation and mechanistic studies. Regardless of study size, new innovations in analyzing PPI datasets will be possible through incorporation of protein structure and interaction prediction tools.
